# The value of narrow-band imaging bronchoscopy in diagnosing central lung cancer

**DOI:** 10.3389/fonc.2022.998770

**Published:** 2022-09-16

**Authors:** Juanjuan Zhu, Rui Liu, Xiancheng Wu, Qin Li, Beilei Gong, Yuanbing Shen, Yurong Ou, Wei Li

**Affiliations:** ^1^ Provincial Key Laboratory of Respiratory Disease in Anhui, Department of Respiratory Disease, The First Affiliated Hospital of Bengbu Medical College, Bengbu, China; ^2^ Center for Clinical Medicine of Respiratory Disease (Tumor) in Anhui, Department of Respiratory Disease, the First Affiliated Hospital of Bengbu Medical College, Bengbu, China; ^3^ Department of Respiratory Disease, Dangshan County People’s Hospital, Dangshan, China; ^4^ Department of Respiratory Disease, The Second Affiliated Hospital of Bengbu Medical College, Bengbu, China; ^5^ Department of Pathology, The First Affiliated Hospital of Bengbu Medical College, Bengbu, China

**Keywords:** narrow-band imaging, central lung cancer, diagnosis, vascular pattern, histological type, CD34

## Abstract

**Aims:**

This research aimed to study the value of narrow-band imaging(NBI) in the diagnosis of central lung cancer.

**Materials and methods:**

This study included 916 patients with clinical suspected of central lung cancer or follow-up of patients after curative lung cancer surgery. All of the patients were examined by Olympus Evis Lucera electronic bronchoscope system, any sites that were abnormal when viewed by white-light bronchoscopy (WLB) or NBI were biopsied, four to six biopsies were taken at each site of the abnormal region visualized as lesions, we record the endoscopic features of NBI and compared with histopathology results, to evaluate the diagnostic value of NBI for central lung cancer and the relationship between vascular patterns of NBI and histological types of lung cancer, and try to establish a multinomial logistic regression model for predicting the histological types of lung cancer. The biopsy specimens were examined by CD34 antibody through immunohistochemistry (IHC) method, CD34 marked microvessel density(MVD), compared the number of microvessels between benign and malignant diseases and the number between different histological types of lung cancer, to verify the results of NBI.

**Results:**

NBI provided high sensitivity (91.7%), specificity (84.9%), positive predictive value (97.6%), negative predictive value (61.5%), and agreement rate (90.7%). The predominant vascular patterns in the well-defined histological types of lung cancer were dotted blood vessels (121 patients), tortuous blood vessels (248 patients), and abrupt-ending blood vessels (227 patients). Logistic regression analysis of the results showed that smoking status of the patient, combined with vascular patterns under NBI, and age partly affect the histological types of lung cancer.

**Conclusions:**

NBI is highly accurate for the diagnosis of central lung cancer.

## Introduction

The global cancer statistics report shows that in 2020, the number of new cases of lung cancer exceeded 2.2 million, and the number of new deaths caused by lung cancer was about 1.8 million, accounting for 11.4% of new cases and 18.0% of deaths from malignant tumors respectively, which means that lung cancer is one of the tumors with the highest morbidity and mortality (1). The situation of lung cancer in China is more severe than that in the world. In 2020, there were about 816,000 new cases of lung cancer and about 715,000 deaths caused by lung cancer in China, accounting for 17.9% of new cases and 23.8% of deaths from malignant tumors, ranking first among malignant tumors (2). The clinical symptoms of early-stage lung cancer are atypical, leading to the fact that most patients are diagnosed at an advanced stage. As such, the early diagnosis of lung cancer is crucial to patient survival but is an ongoing challenge to accomplish. Thus, improving technologies for accurate, early-stage lung cancer diagnoses is sorely needed (3).

The occurrence and development of lung cancer occurs *via* a gradual evolution from the normal lung cell phenotype to cancerous cells. According to the different pathological types, lung cancer is divided into non-small cell lung cancer (NSCLC) and small cell lung cancer (SCLC), of which NSCLC accounts for 85% of all lung cancer (4). Under the premise of pathological diagnosis as the gold standard, although there are many diagnostic methods for early lung cancer, bronchoscopic biopsy is still the most effective diagnostic technique for central lung cancer (5). As early lung cancer under WLB are nonspecific and have a high rate of missed diagnosis, new endoscopic techniques, such as fluorescence bronchoscopy and NBI, may be helpful in improving diagnostic accuracy, especially at the early stage (6).

Within these new diagnostic methods, fluorescence bronchoscopy, which uses exogenous fluorescent substances or tissue auto-fluorescence imaging, has high sensitivity 0.92 vs 0.70 (WLB) but relatively poor specificity 0.67 vs 0.78 (WLB) (7). NBI, by contrast, has more potential to achieve high sensitivity and specificity, and preliminary clinical data has highlighted this potential for early stage cancer diagnoses and the development of new therapeutics (8). More researches on the use of NBI for early stage lung cancer diagnosis are needed.

NBI uses a special filter to remove the broadband light in the RGB (red-green-blue) spectrum, leaving only narrow band spectrum (blue and green). Once the narrow band is left, the blue narrow band light is used to visualize capillaries of the surface mucosal layer; and the green narrow band light can be used to visualize the thick blood vessels inside the mucous membranes (9). The advantages of NBI for lung cancer diagnoses is that it can be used to simultaneously image the morphology of bronchial mucosa and the epithelial vascular network. The combination of these two bands creates excellent contrast on the mucosal surface, reduces examination time, and eliminates non-productive biopsies (10, 11).

NBI was originally used in the diagnosis of digestive system diseases, as well as for the diagnosis of head, neck, and urinary tumors (9). NBI has been shown to have high accuracy of magnifying NBI in distinguishing between cancerous and noncancerous gastric lesions, with a meta-analysis by Zhou et al., showing a sensitivity of 0.88 and specificity of 0.96, the area under the curve (AUC) was 0.97 (12). Additionally, a meta-analysis of NBI for the diagnosis of primary nasopharyngeal carcinoma showed that the diagnostic sensitivity and specificity of NBI were 90%and 95%, AUC was 0.98 (13).

The application of NBI to lung imaging benefits from a synergistic combination with high magnification bronchoscopy (11, 12, 14). Such bronchoscopy is used to distinguish between benign and malignant lesions in the airway, which allows lesions to be identified that are not easily found *via* routine endoscopy (15). Although NBI has been studied for the diagnosis of precancerous lesions and early central lung cancer those reports were from single-center small studies with a small number of patients (16, 17). Despite these limitations, the reported sensitivity and specificity of NBI was high. NBI has additional potential therapeutic benefits through its use in evaluating tumor extensions and can significantly influence therapeutic decision making for lung cancer treatment (18).

Up to now, few studies have determined how vascular patterns observed *via* NBI are related to the histological phenotype of lung cancer (19). Of note, Shibuya et al. defined three types of pathological vascular patterns in premalignant and malignant lesions of bronchial mucosa: dotted blood vessels, tortuous blood vessels, and abrupt-ending blood vessels (20). An additional study by Zaric et al. showed that: dotted blood vessels were identified in 68.4% of adenocarcinoma and 31.6% of SCC; tortuous blood vessels were identified in 72% of SCC, 8% of adenocarcinoma, and 12% of SCLC; and abrupt-ending vessels were identified in 81% of SCC, 14.3% of SCLC and 4.8% of adenocarcinoma (21). These differences in the vascular patterns provide a mechanism by which NBI can be used to differentiate between adenocarcinoma and SCC. In a related study, Miyazu and co-workers showed that NBI could identify several vascular and mucosal patterns, including intraepithelial capillary papillary loops (IPCL), abnormal tumor vessels, extremely increased fine vascular networks, and an erythematic and cobblestone appearance of the mucosa (19). The fact that IPCL pattern was identified in 30 of 38 patients with SCC, and the cobblestone appearance was found in 11 of 15 patients with adenocarcinomas indicates that vascular patterns visible using NBI display some predictive value for differentiating types of lung cancer. However, the two studies mentioned above observed that vascular patterns visualized by NBI may be only related to the histological types of lung cancer. Methodological weaknesses in the aforementioned studies were also noted, including the small sample size of one study, the retrospective and non-randomized nature of the other, and lack the support of an accurate mathematical model to explain the data. As a result, the need for more studies regarding the use of NBI for lung cancer diagnosis and imaging remain.

The purpose of the study reported herein was to evaluate the value of NBI in the diagnosis of central lung cancer, and to analyze the relationship between the 3 vascular patterns observed in lung cancer and the different histological variants using multivariate polynomial logistic regression analysis. Compared to previously reported studies on this topic, this study has significant novelty in its use of a randomized study design, a large number of study participants, the use of an improved bronchoscopy device, and the fact that many more biopsies were taken.

## Materials and methods

### Patients

The study was conducted at a dedicated respiratory endoscopy unit in the First Affiliated Hospital of Bengbu Medical College, located in Anhui province in China. And the study conducted between May 2015 and December 2019. All of the patients screened for the enrollment were previously scheduled for routine bronchoscopy due to a high probability of lung cancer, or were patients who had been designated for follow up screenings following curative lung cancer surgery. The study was approved by the hospital ethics committee (Approval Number 2015-017), following standard review procedures. Inclusion criteria for participant enrollment in the study were (1): age over 18 years and a reasonable suspicion of lung cancer based on chest CT imaging (i.e. observation of bronchial stenosis involving the lung segment, lobe, or whole lung atelectasis; pulmonary hilar nodule or mass, etc.) (2); patients had one of the following clinical symptoms: hemoptysis of unknown origin, irritable cough of unknown origin, hoarseness of unknown origin, localized wheezing sound of unknown origin (3);surveillance of patients after curative lung cancer surgery and evaluation of known lung cancer were followed up regularly. Exclusion criteria from the study were (1): pregnancy of a potential participant (2); the participant’s refusal to participate in the study (3); the participant’s inability to tolerate and/or cooperate with the bronchoscopy procedure (4); participant’s known or suspected allergy to anesthetics or other medications (5); other medical conditions that presented within the last three months, including heart failure, unstable angina, myocardial infarction, cerebral infarction or cerebral hemorrhage (6); participants with longer-term health concerns, including severe coagulation disorder, uncontrolled arrhythmia, and hypertension (7); participant was suspected of concomitant aortic aneurysm (8); participant had active massive hemoptysis.

### Research design and methods

All of the patients must have had a chest CT scan, routine blood test, ECG, bleeding and coagulation function test, and blood test for infectious agents (including syphilis antibodies, HBsAg, anti-HBs, HBeAg, anti-HBe, anti-HBc, HCV, HAV, HEV, HIV). All patients were required to fast from food and water for not less than eight hours before the start of the procedure. All participating patients were informed about the procedure prior to undergoing it, the potential benefits and risks of the specific procedure and of the overall purpose of the study, and all had signed an institutional informed consent form, with details on the intake form including the image, age, gender, smoking history, biopsy sites, contact information, and tracking of the pathologic results.

### Pre-procedure preparations

Anesthesia took place *via* nasal vestibular with cotton yarn soaked in solutions of adrenaline and lidocaine. Following this, 10 mL of 2% lidocaine was used for the mask airway anesthesia, with lidocaine local anesthetic spray used in the throat during the procedure. First attempts to obtain successful bronchoscopies were conducted *via* nasal passage, with a backup option of using the mouth to access the airway if necessary. Intraoperative monitoring of the participants included: heart rate monitoring, non-invasive arterial blood pressure monitoring, oxygen saturation value monitoring and the use of a simulated ECG. Bronchoscopic equipment used in this study was the BF-H290 NBI video bronchoscope and EVIS LUCERA SPECTRUM (CV-260SL) video processor unit. Video bronchoscopy images were displayed on a 19-inch OEV-191LCD monitor and endoscopic images were acquired and saved on the medical imaging system. The examination of the tracheobronchial tree started with WLB, followed by NBI. Once the pathological sites were identified under WLB or NBI, targeted biopsies were performed to obtain between four and six tissue specimens for pathological examination (using a single pair of forceps per site to avoid the possibility of inter-site cross-contamination). If multiple lesions were found in the same patient, the most distal lesions were biopsied before the proximal lesions, in order to prevent bleeding from the biopsy sites which might blur the field of vision. The NBI analysis was performed at the biopsy site during the procedure and recorded separately. The patient’s general information and the location and quantity of biopsy specimens were provided to the pathologist.

### Classification criteria

In this study, we used consensus between two or three experienced bronchoscopists in order to determine the predominant vascular pattern in the observed lesion. Pathological vascular patterns were identified by the Shibuya descriptors (dotted blood vessels, tortuous blood vessels, and abrupt-ending blood vessels).

### Histopathological analysis

The pathological results were evaluated by two experienced pathologists independently, with disagreements resolved by consensus. Both pathologists were blinded to the bronchoscopic findings and to the biopsy sites. Histological and/or cytological findings were used for definitive lung cancer diagnosis, with cancer-free patients scheduled for a follow-up appointment in six months. In this study, all of the bronchoscopic biopsy specimens with HE staining were initially screened by pathologists. 71 case samples were screened, then stained with Anti-CD34 antibody (antibody clone QBEnd/10) using IHC. This was done to label microvessel density according to the Weidner correction method which enables counting of the microvessels in benign and malignant lesions and different histological types of lung cancer (22). Counting of all specimens were performed by senior pathologists.

### IHC procedure

The paraffin-waxed specimens were sliced into 4-μm sections and analyzed by IHC(EliVision Plus IHC Kit; Fuzhou Maixin Biotechnology Development Co., China). Briefly, specimens were first deparaffinized with xylenes, which dissolved the paraffin coating. Then, antigen retrieval was performed in a citrate buffer (pH 6.0) at room temperature, over a 15 minutes time frame. Following that retrieval, endogenous peroxidase activity was blocked by incubation of the samples in a 3% hydrogen peroxide in methanol solution at room temperature for 10 minutes. Then, the primary antibody, a murine anti-human CD34 polyclonal antibody (50 μL, diluted 1:200 with deionized water, obtained from Abcam, USA), was applied and the samples were stored overnight at 4°C. As a negative control, 50 μL of phosphate-buffered saline (PBS, buffered at pH 7.4) was used in lieu of the 50 μL of primary antibody. Following overnight freezing, specimens were warmed at 37°C for 30 minutes, washed with PBS three times for 5 minutes each, and then treated with 50 μL of a polymer enhancer for 20 minutes in a 37°C incubator. Specimens were washed with PBS three times for five minutes each and then treated with 50 μL of a polymerized horseradish peroxidase-anti-mouse immunoglobulin G solution (IgG) (DAB Kit; Maixin Biological Co., China) for 30 minutes in a 37°C incubator. Reaction products were visualized using a 3’-diaminobenzidine stain (DAB Kit; Maixin Biological Co., China). Sections were counterstained with H&E, dehydrated, and observed under light microscopy. The percentage of positive cells was calculated by dividing the number of positive tumor cells by the total number of counted tumor cells and multiplying by 100%.

### Principle of microvessel counting by IHC

Any individual brown vascular endothelial cell or cell cluster stained by CD34, which is visibly separated from adjacent microvessels, tumor cells, and other connective-tissue elements is considered a single, countable microvessel. Vessel lumens, although usually present, are not necessary for a structure to be defined as a microvessel, and red cells are not used to define a vessel lumen. A number of features were not included in the microvessel count, including: microvessels in sclerotic areas within a tumor, where are areas that typically contain low microvessel concentrations; microvessels in the soft tissue at the junction of the tumor; the thick smooth muscle wall; and the diameter of the lumen > 8 red blood cells. The areas of highest neovascularization were found by scanning the tumor sections at low power (100X). We selected three areas that showed clear staining, good background control, the most densely packed microvessels and the largest distribution of tumor cells, counted the stained microvessels at high power (400X), and took the average value obtained from the three areas as the MVD value.

### Statistical analysis

Data analysis was done using SPSS 17 Version (SPSS). Continuous variables were described using means and standard deviations (SDs), independent sample t tests and single factor variance analysis used for statistical computing. Categorical data were described as a percentage (%) and chi square test. The categorical variables of adenocarcinoma, SCC and SCLC were more challenging to account for, as the traditional binary logistic regression analysis would ignore relevant information and increase the risk of type I errors. Therefore, the relationship between the vascular patterns and histology types of lung cancer was analyzed by assigning the histological classification of lung cancer as the dependent variable, with gender, age, smoking status, and vascular patterns as independent variables. We used SPSS software for multinomial logistic regression analysis. A p-value (*p*< 0.05)was used to indicate as a statistically significant difference.

## Results

A total of 916 patients were included in this study. The baseline characteristics of the patients are summarized in **Table 1** There were 760 male patients (82.97% of the total participant population) and 156 female patients (17.03%). The ages of the participants ranged from 19 to 87 years old, with an average age of 64.18 ± 9.771years. 636 of the patients (69.43%) were smokers and 280 patients (30.57%) never smoked. There were 355 (38.75%) cases with the main symptoms of phlegm blood or hemoptysis, 302 (32.97%) cases of cough, expectoration, fever, 140 (15.28%) cases of chest tightness, shortness of breath or chest pain), 38 (4.15%) cases were found in physical examination, 36 (3.93%) cases of hoarseness, 15 (1.64%) cases of anorexia, vomiting, weight loss, 9 (0.98%) cases of superior vena cava obstruction, 8 (0.87%) cases of headache and dizziness, 6 (0.66%) cases of low back pain, 4 (0.44%) cases of cervical mass, 3 (0.33%) cases of leg numbness or weakness. 605 (66.05%) cases were mainly manifested as pulmonary hilar mass in chest CT, 183 (19.98%) cases of obstructive atelectasis or obstructive pneumonia, 76 (8.30%) cases of lung hilar nodule, 36 (3.93%) cases of bronchial stenosis, 16 (1.74%) cases of pleural effusion.

**Table 1:** Clinical data of all patients.

Characteristic	All, No. of patients (%)
Age, median (range), years	66 (19-87)
Sex	
male	760 (82.97%)
female	156 (17.03%)
Smoking status	
smoker	636 (69.43%)
never smoker	280 (30.57%)
Imaging manifestation	
pulmonary hilar mass	605 (66.05%)
obstructive atelectasis/pneumonia	183 (19.98%)
lung hilar nodule	76 (8.30%)
bronchial stenosis	36 (3.93%)
pleural effusion	16 (1.74%)
Diagnosis	
malignant tumors	790 (86.24%)
inflammation	85 (9.28%)
tuberculosis	13 (1.42%)
squamous metaplasia/intraepithelial lesion	11 (1.20%)
tuberculous pleurisy	6 (0.65%)
sarcoidosis	5 (0.55%)
fibroadenoma	3 (0.33%)
organizing pneumonia	2 (0.22%)
pulmonary aspergilloma	1 (0.11%)

### Pathological results and classification

A total of 916 patients were examined by bronchoscopy, with all of them accurately diagnosed. 790 cases of them were diagnosed with malignant tumors, benign lesions including 85 cases of inflammation, 13 cases of tuberculosis, 11 cases of squamous metaplasia or squamous intraepithelial lesion, 6 cases of tuberculous pleurisy, 5 cases of sarcoidosis, 3 cases of fibroadenoma, 2 cases of organizing pneumonia, 1 case of pulmonary aspergilloma. The most common histopathology types of lung cancer were: SCC 293 cases (including 28 cases of squamous cell CIS), SCLC 222 cases, adenocarcinoma 136 cases, 77 cases of NSCLC, 57 cases of poorly differentiated carcinoma. The less common histopathology types of lung cancer were: 3 cases of CIS (undifferentiated), 1 case of large cell lung cancer, 1 case of mucoepidermoid carcinoma. The most common lung cancer lesion sites were right upper lobe 221 (27.97%) cases, left upper lobe 190 (24.05%) cases, left lower lobe 111(14.05%) cases, left principal bronchus 78 (9.87%) cases, right lower lobe 60 (7.59%) cases (see **Table 2**).

**Table 2:** Localizations of lung cancer under NBI.

Localization	Frequency	Percent (%)
Right main bronchus	34	4.30%
Right upper lobe bronchus	221	27.98%
Right intermediary bronchus	49	6.20%
Right middle lobe bronchus	47	5.95%
Right lower lobe bronchus	60	7.60%
Left main bronchus	78	9.87%
Left upper lobe inherent segment bronchus	146	18.48%
Left upper lobe lingual segment bronchus	44	5.57%
Left lower lobe bronchus	111	14.05%
Total	790	100%

### The value of NBI in the diagnosis of central lung cancer

**Figure 1:**
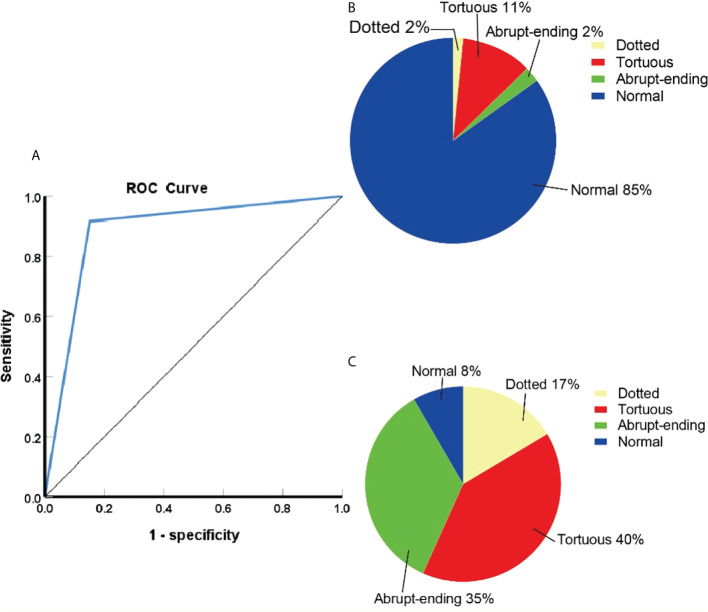
**(A)**ROC curve of NBI in the diagnosis of central lung cancer. **(B)**The proportion of different vascular patterns in benign lesions. **(C)**The proportion of different vascular patterns in lung cancer.

NBI diagnoses were positive in 742 cases and negative in 174 cases. Compared with the pathological results, the diagnostic sensitivity, specificity, positive predictive value, and negative predictive values were 91.7%, 84.9%, 97.6%, and 61.5%, respectively. The accuracy of diagnosis was 90.7%, AUC was 0.88 (95%CI:0.85-0.92). The differences between the detection of benign and malignant lesions was quantified using a chi square test, which gave an χ^2^ value of 25.988, *p*< 0.05, indicating a statistically significant difference. A comparison of the NBI findings and pathological results can be seen in **Table 3**, with the ROC curve of NBI in the diagnosis of central types of lung cancer shown in **Figure 1A** and a summary of the composition of vascular patterns in benign and malignant lesions in **Figures 1B, C**.

**Table 3:** NBI video bronchoscopy comparison with pathological results.

NBI	Pathological results		Total	χ2	*p*
	Malignancy	Benign			
+(Abnormal)	724	19	742	25.988	<0.001
-(Normal)	66	107	174		
Total	790	126	916		

### Results of NBI

**Figure 2:**
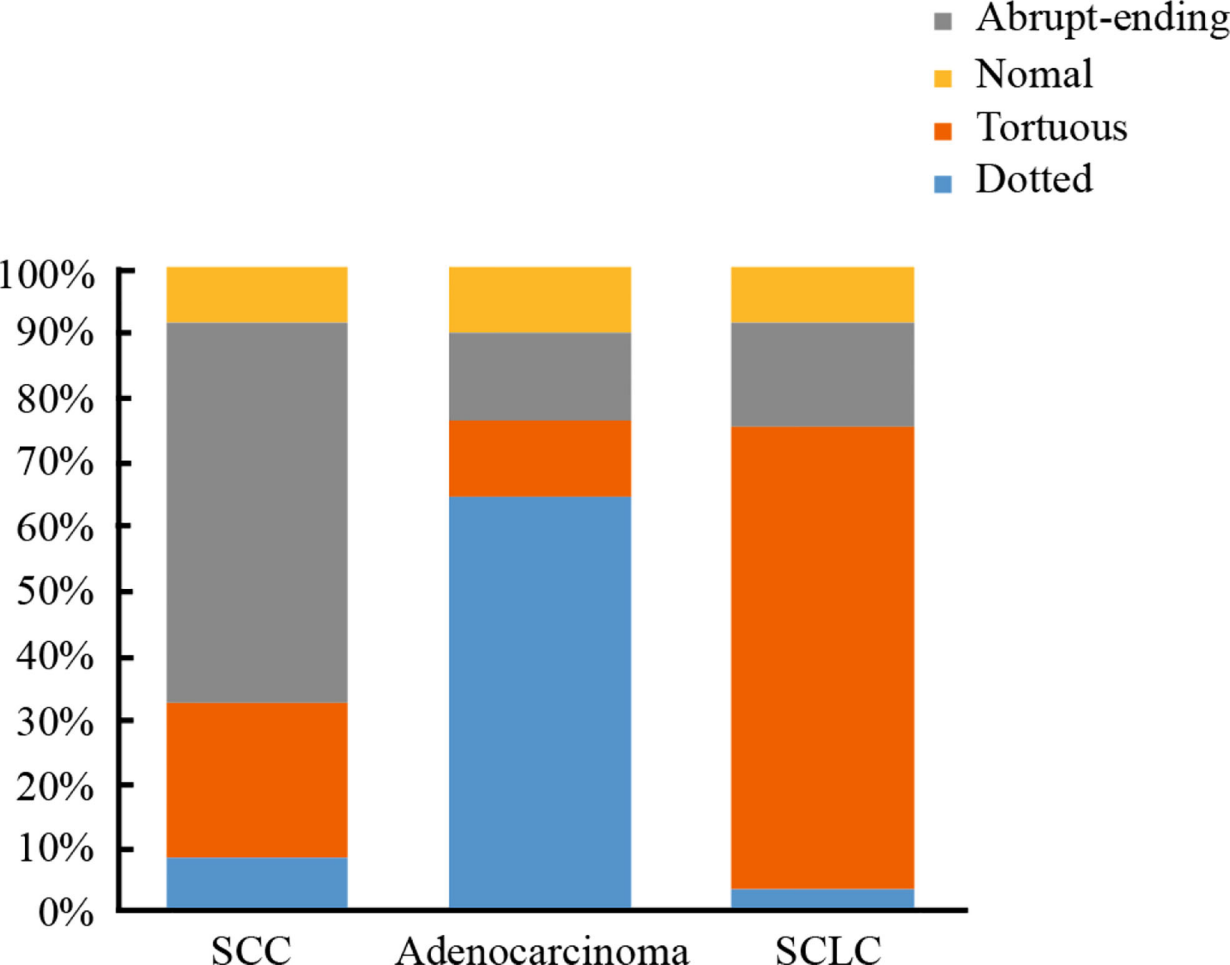
The proportion of vascular patterns in different histological types of lung cancer.

Of the 916 cases that underwent NBI bronchoscopy, 742 cases were classified as positive according to NBI, of which 724 cases were diagnosed as lung cancer. 174 cases were negative according to NBI analysis, of which 66 patients were diagnosed as lung cancer. Within the positive lung cancer diagnoses, a total of 651 cases were classified as lung cancer with clear histological types: 293 cases of SCC, 222 cases of SCLC, and 136 cases of adenocarcinoma. Classifying the lung cancer diagnoses by blood vessels types led to the following distribution pattern: dotted blood vessels was the most predominant type of pathological blood vessels in 121 patients, tortuous blood vessels was the most predominant type of pathological blood vessels in 248 patients, 227 patients had abrupt-ending blood vessels, and 55 patients had no abnormal blood vessels (24 cases were SCC, 18 cases were SCLC, 13 cases were adenocarcinoma) (see **Table 4**). The abrupt-ending vascular pattern was more common in SCC than other vascular types, representing 76.2% of the positive cases investigated; the dotted vascular pattern was more common in adenocarcinoma compared to other vascular types, with 72.7% of the positive adenocarcinoma cases showing this pattern; and the tortuous vascular pattern was more common in SCLC, representing 64.9% of the positive lung cases in this category (see **Figure 2**). The positive diagnosis rates using NBI video bronchoscopy of SCC, SCLC, and adenocarcinoma were 91.8%, 91.9%, 90.4%, respectively.

**Table 4:** Relation between histological types of lung cancer and visual appearance of pathological vascularization under narrow band imaging (NBI) video bronchoscopy.

Histological types of lung cancer	Visual appearance			Total N (%)
	Dotted blood vessels N (%)	Tortuous blood vessels N (%)	Abrupt-ending blood vessels N (%)	
SCC	25 (20.66%)	71 (28.63%)	173 (76.21%)	269 (45.13%)
Adenocarcinoma	88 (72.73%)	16 (6.45%)	19 (8.37%)	123 (20.64%)
SCLC	8 (6.61%)	161 (64.92%)	35 (15.42%)	204 (34.23%)
Total	121 (100%)	248 (100%)	227 (100%)	596 (100%)

### Logistic regression analysis

After assigning the variables of gender, age, smoking status, and vascular pattern to the patient data (see **Supplementary Table S1**), univariate polynomial logistic regression analysis was carried out for each of the above four variables in succession (see **Supplementary Tables S2, S3**). Using adenocarcinoma as a reference category for all cases, the occurrence of SCC in women was 0.062 (Exp (B)) times of men (*p*< 0.001), the incidence of SCLC, females were 0.432 (Exp (B)) times of males (P=0.002); the occurrence of SCC (*p*=0.006) were 1.779 (Exp (B)) times higher in the high grade age group than in the low grade age group, the occurrence of SCLC (*p*=0.068) were no statistically significant difference among age groups; the occurrence of SCC in smoking patients was as much as 19.204 times of non-smoking patients (*p*< 0.001), the occurrence probability of SCLC, smoking patients were as much as 8.104 times of non-smoking patients (*p*< 0.001); the probability of SCC showing abrupt-ending blood vessels was 32.051 times that of showing dotted blood vessels (*p*< 0.001); the presence of tortuous blood vessels was 15.620 times that of dotted blood vessels (*p*< 0.001); and the probability of SCLC showing abrupt-ending blood vessels was 20.263 times that of showing dotted blood vessels (*p*< 0.001), tortuous blood vessels was 110.688 times that of dotted blood vessels. Our univariate analysis found that gender, smoking status, and vascular patterns affect the histological types of lung cancer, the age partly affects the histological types of lung cancer. Multivariate polynomial logistic regression analysis was conducted using age, gender, smoking status, and vascular pattern as variables (see **Supplementary Tables S4, S5**), when age, gender and smoking status remain fixed, with results of this analysis demonstrating that the vascular pattern had a significant influence on the histological types of lung cancer observed. Based on **Supplementary Tables S4, S5** we conclude that using adenocarcinoma as a reference, the equation of NBI predicting SCC was:


$$logit\left(\pi_{2}/\pi_{1}\right)\;=\;-\;\left(-0.971\right)\;-0.993\;X_{1}-0.435\;X_{2}+2.820\;X_{3}+3.589\;X_{41}+2.925\;X_{42}$$

The equation of NBI predicting SCLC was: *logit* (*π*_3_/*π*_1_) = − (−3.479) +0.956 *X*_1_−0.742 *X*_2_+2.913 *X*_3_+3.158 *X*_41_+4.916 *X*_42_ Based on this data, we can conclude that compared with adenocarcinoma, the risk of abrupt-ending blood vessels in SCC was 36.196 times that of dotted blood vessels, with the risk of tortuous blood vessels 18.638 times that of dotted blood vessels. The risk of abrupt-ending and tortuous blood vessels in SCLC were 23.526 and 136.415 times that of dotted blood vessels. This means that abrupt-ending vascular pattern occur frequently in SCC-type lung cancer, whereas the tortuous vascular pattern is more likely to occur in SCLC. The overall prediction accuracy of regression model was 70.5%.

### IHC results

**Figure 3:**
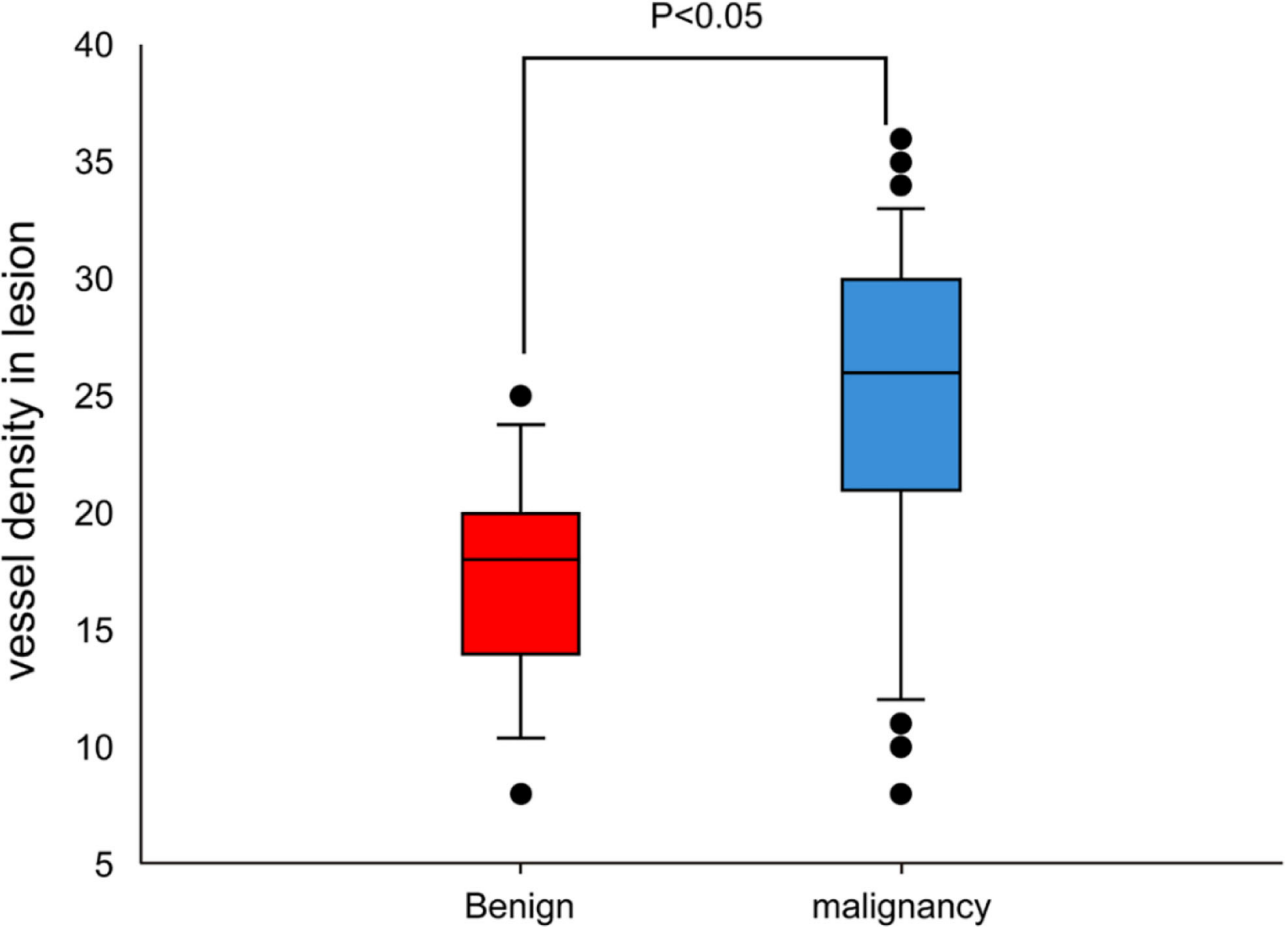
Comparison of MVD value between benign and malignant lesions.

**Figure 4:**
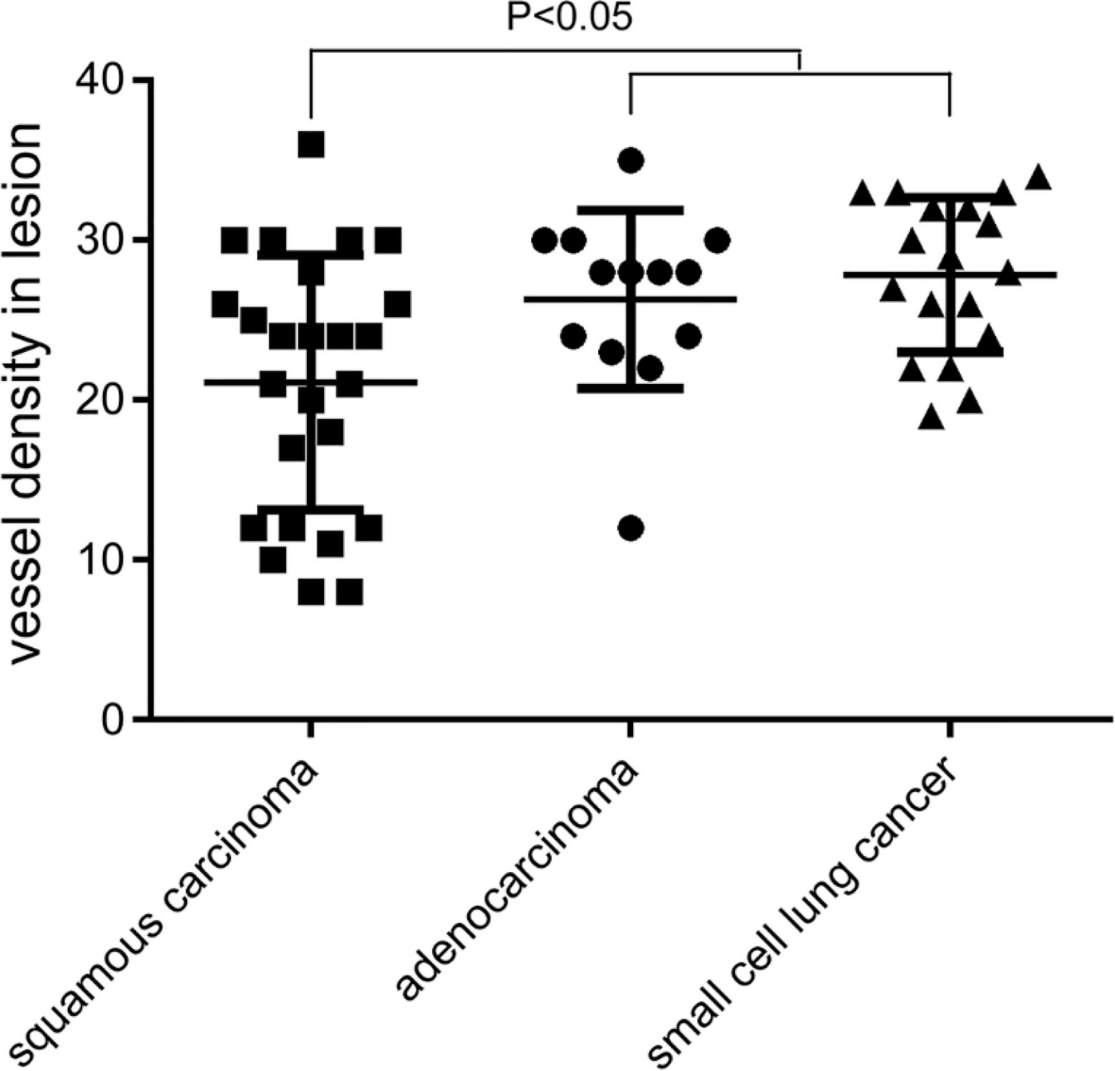
Comparison of MVD value for SCC, adenocarcinoma, and SCLC.

**Figure 5:**
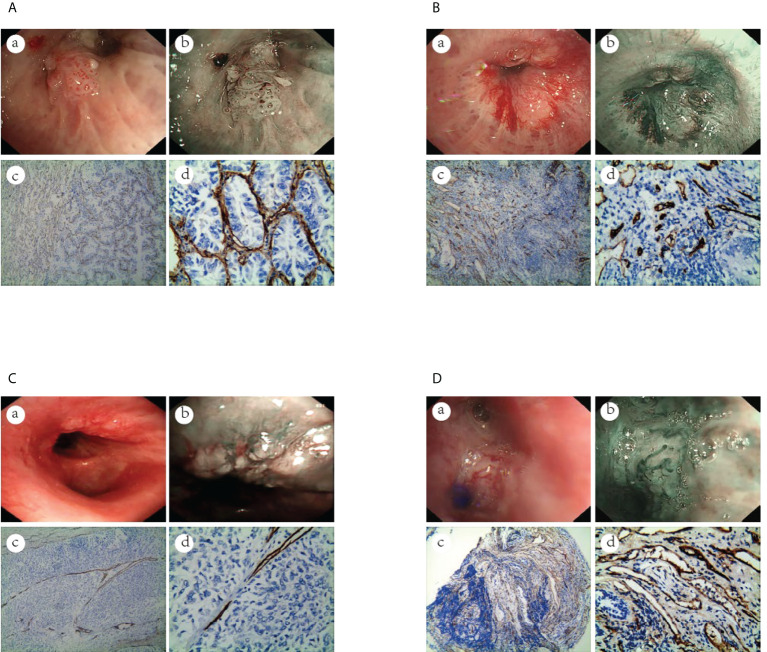
The vascular images of WLB, NBI and histological specimens after IHC for malignant lesions. **(A)** Dotted blood vessels in adenocarcinoma of the lung (a) WLB image. (b) NBI shows that there are obvious dotted blood vessels. (c, d) IHC staining of the tissue specimen shows that there are obvious dotted blood vessels(the scale of Figure c is 100μm, the scale of Figure d is 50μm). **(B)** Tortuous blood vessels in SCC of the lung (a) WLB image. (b) NBI shows that there are obvious tortuous blood vessels. (c, d) IHC staining of the tissue specimen shows that there are obvious tortuous blood vessels(the scale of Figure c is 100μm, the scale of Figure d is 50μm). **(C)** Abrupt-ending blood vessels in SCC of the lung (a) WLB image. (b) NBI shows that there are obvious abrupt-ending blood vessels. (c, d) IHC staining of the tissue specimen shows that there are obvious abrupt-ending blood vessels(the scale of Figure c is 100μm, the scale of Figure d is 50μm). **(D)** Tortuous blood vessels in SCLC of the lung (a) WLB image. (b) NBI shows that there are obvious tortuous blood vessels. (c, d) IHC staining of the tissue specimen shows that there are obvious tortuous blood vessels (the scale of Figure c is 100μm, the scale of Figure d is 50μm).

**Figure 6:**
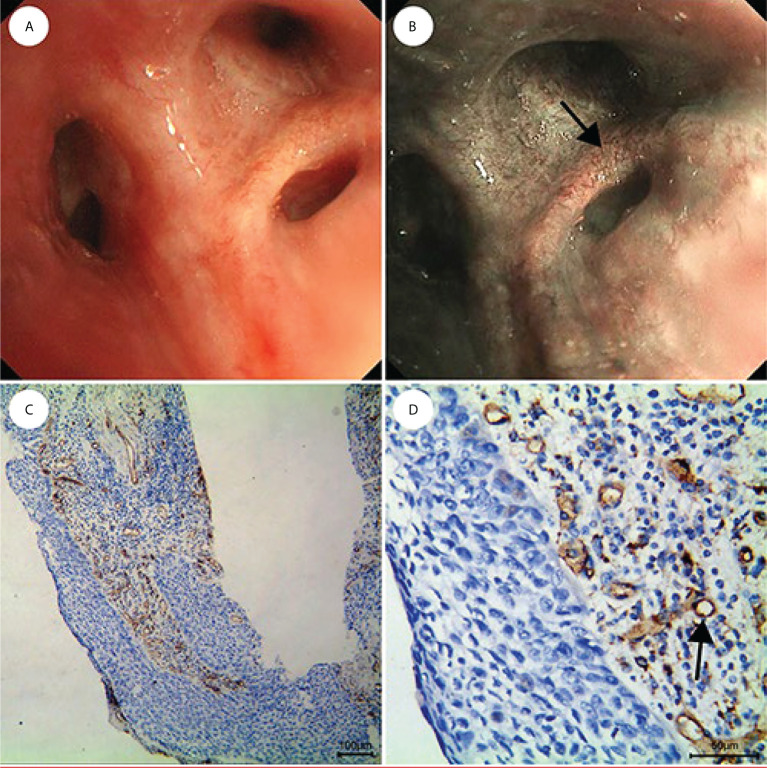
CIS is shown as lesions with only dotted blood vessels, small spiral or screw type tumor vessels, and a few tortuous vessels under NBI. **(A)** WLB image. **(B)** NBI shows that there are a few dotted, small spiral or screw type, and tortuous vessels. **(C, D)** IHC staining of the tissue specimen shows that there are obvious tortuous blood vessels [the scale of **(C)** is 100 μm, the scale of **(D)** is 50 μm].

**Figure 7:**
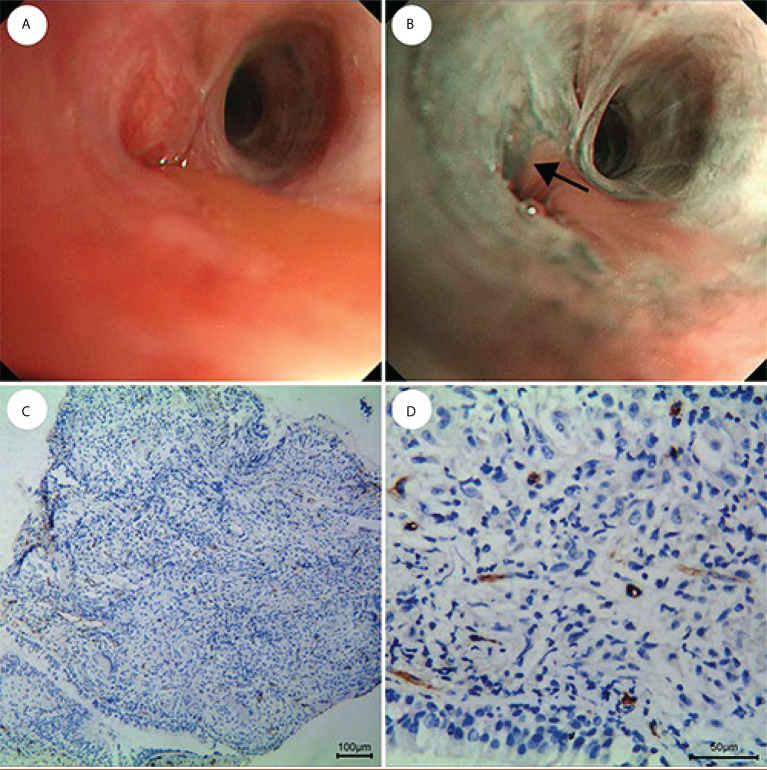
Fewer and regular blood vessels in inflammation of the lung. **(A)** WLB image. **(B)** NBI shows that there are fewer and regular blood vessels. **(C, D)** IHC staining of the tissue specimen shows that there are fewer and regular blood vessels [the scale of **(C)** is 100 μm, the scale of **(D)** is 50 μm].

The MVD value as determined by anti-CD34 was 24.5 ± 7.2 inches for the malignant lesions (n=56), compared with 17.3 ± 4.5 inches for the benign lesions (n=15) (*p*< 0.001) (see **Figure 3**). The MVD value of SCLC (n=18), SCC (n=25) and adenocarcinoma (n=13) cancer subtypes were 27.8 ± 4.8, 21.1 ± 8.0, and 26.3 ± 5.6 inches, respectively. The MVD value in SCLC patients and adenocarcinoma patients were higher than in those of the SCC patients, with statistically significant differences obtained (*p* =0.002, *p* =0.024, respectively) (see **Figure 4**). There was no statistical difference between the MVD value observed in SCLC and adenocarcinoma cancer (*p* =0.527). Examples of the vascular images of WLB, NBI and histological specimens after IHC for both benign and malignant lesions can be seen in **Figures 5**–**7**, with clear differences in the types of lesions noted.

### Complications

The complications observed through the use of NBI were equivalent to those observed when using routine bronchoscopy, and include nose bleeds, throat spasms, post-procedure bloody sputum or hemoptysis, chest tightness, post-procedure fever, patient sensitivity to the anesthetic, cerebral hemorrhage, arrhythmia, pneumothorax, sudden death and so on. In this study, only 51 patients experienced chest tightness postoperatively, which improved after oxygen inhalation and the use of bronchial spasm drugs. 12 patients developed headache and fatigue, which improved after rest. 205 patients experienced post-procedure bloody sputum, and all recovered spontaneously without additional serious complications.

## Discussion

Compared with cervical cancer and breast cancer, the early diagnosis of lung cancer is very challenging (23). Although studies show that the death rate of lung cancer can be reduced through early stage diagnosis, such as through using low-dose computed tomography (LDCT) imaging, such imaging includes the potential for negative side effects from the radiation of the CT, as well as potential challenges due to overdiagnosis and overtreatment of small lesions (24–26). Furthermore, because of a lack of tissue specimens, LDCT needs to be combined with other imaging and/or detection methods, and unfortunately, the early detection rate of central lung cancer remains low (27). Other options such as a special light bronchoscopy technique has been used, which has advantages in terms of high sensitivity and minimal level of invasiveness to patients (28, 29). A meta-analysis of 10 studies by Sun et al. in 2020 evaluated the value of AFB compared with WLB in the detection of bronchial cancers (8). The sensitivity of AFB was higher than that of WLB, but the specificity was lower than WLB. Therefore, the emergence of NBI imaging has the potential to address/remediate this deficiency.

The NBI system reduces unnecessary intermediate colors, provides better contrast on the surface of mucosa, reduces physiological damage and overall examination time, and eliminates non-productive biopsies. Combined with the high magnification of the video bronchoscopy system, NBI can more clearly show the microvascular architecture of the bronchial mucosa. Moreover, NBI has additional practical advantages in terms of providing information in real time, lowering the overall cost of diagnosis, and enabling minimally invasive diagnostic techniques.

Studies showed that NBI has a high sensitivity and specificity in the diagnosis of lung cancer that is superior to AFI and WLB (30, 31). For example, a meta-analysis of eight studies of NBI bronchoscopy in the detection of premalignant airway lesions showed that NBI had a higher sensitivity and specificity compared with AFB (17). Combining AFB and NBI did not improve the test performance significantly, although significant heterogeneity among the studies was observed. Our study results, reported herein, also found that NBI was of great value in the diagnosis of central lung cancer, with a sensitivity and specificity of 91.7% and 84.9%, respectively. A study by Vincent et al. of 22 patients with known or suspected bronchial dysplasia or malignancy found a sensitivity ratio of 1.63 for the combination of NBI and WLB compared to the use of WLB alone, which had a specificity for dysplasia and cancer of 64% (32). It is noteworthy that NBI detected 5 instances of dysplasia or cancer (23% of patients) that were not detected with WLB. Therefore, the authors determined that NBI is helpful in improving the diagnostic rate of early lung cancer, but caution that the small size of the study population can complicate general applicability of the results obtained. An even larger study by Chen et al. of 153 patients who were highly suspected of central lung cancer found the sensitivity of NBI in the diagnosis of central lung cancer to be 63.5%, whereas the specificity was 75.0% (8). In this scenario, the diagnostic value was slightly lower, perhaps due to the bronchoscopy devices used in this study (Japanese Olympus BF-1T260), which are known to have somewhat limited definition.

The diagnostic value of our study was higher than that reported in the literature. We believe this may be due to several things, for the first time, of an Olympus BF-H290 as a microscopy device. Furthermore, the discrimination power of the vascular pattern is more accurate, and we used a greater number of biopsy (4-6 block tissues). Moreover, the proportion of invasive lesions detected was slightly higher than the proportions determined in some of the above-mentioned studies. In our study, we found 31 cases of CIS, of which 28 cases were SCC CIS.CIS appeared as lesions with only a few dotted, small spiral or screw type and tortuous vessels under NBI, consistent with the study results reported by Shibuya et al. (20). Although the number of cases were small, that study demonstrated the potential for NBI’s use in identifying precancerous lesions. The study of Herth et al. enrolled 62 patients who were at high risk of lung cancer in order to evaluate the diagnostic potential of NBI in airway neoplasia at a pre-invasive stage(moderate to severe dysplasia or CIS) (33).The sensitivity of NBI was 0.53 and the specificity was 0.90.The specificity in this study was similar to our own, but the sensitivity was low, perhaps because only lesions observed by bronchoscopy that met the criterion of “suspicious for intraepithelial malignancy” was classified as a positive finding for pre-invasive neoplasia. Furthermore, the sensitivity of AFI in the diagnosis of intraepithelial neoplasia was 0.65 and the specificity was 0.40. Although the sensitivity of AFI was similar to that of NBI, but the specificity of NBI was significantly increased. The sensitivity of using a combination of AFI and NBI increased to 0.71, although the specificity did not improve. These study results show significant benefits to the use of NBI, either alone or in combination with AFI, which is likely to maximize the observed detection rate.

Additional advantages to using a combination of two imaging techniques is that this combination can observe the mucosal lesions as well as changes of the microvessel in the mucosa. This can make up for the shortcomings of a single technology. The combination of the two technologies will be the focus of screening of precancerous lesions in future. In our study, we did not find moderate or severe dysplasia which was observed in a number of other studies, perhaps because we enrolled patients who were suspected of central lung cancer or who were being followed up after curative lung cancer surgery.

In our study, the IHC method was used for the first time to detect the MVD value of NBI biopsy specimens. Research has shown that anti-CD34 performs better than anti-CD105 or anti-CD31 for the staining of microvessels in lung tissue, and as such, we used anti-CD34 to mark the new blood vessels in tumors. Of note, malignant tumors contained many disordered, irregular microvessels with significant size variation. Benign lesions had more regular microvessels, with a fewer number of vessels and smaller average diameter. Of note, statistically significant differences in the MVD value between benign and malignant lesions were observed. Thus, our results show for the first time that NBI has a special advantage in differentiating benign and malignant lesions based on differences in histology. There are few articles about the correlation between vascular patterns and histological types, a research of high-definition (HD+) bronchoscopy with image enhancement techniques (i-scan) detected more vascular abnormalities correlation with pathology show that abrupt-ending or dotted vessels on these additional lesions are indicative of malignant behaviour and biopsies should be taken. Study confirms that in SCC abrupt-ending vessels are more frequently found but dotted vessels were equally scored in adenocarcinoma and SCC, this is in part consistent with our research (15). A new study screening 78 patients with suspected lung cancer, NBI bronchoscopy revealed a dilated tortuous vascular pattern in 54.8% of the patients, this is consistent with our research, but the conclusion is no relationship exists between NBI vascular patterns and the histology types of lung cancer (34). Adenocarcinoma, SCC, and SCLC are biologically, totally distinct tumors, therefore, predicting the histological types by vascular patterns will have an important impact on the development of therapeutic strategies. Although the MVD counting method is the gold standard for the calculation of tumor neovascularization, it is complex, and the results may not represent the whole tumor angiogenesis. Furthermore, such counting requires static position of the MVD, and therefore it is difficult to execute this method in a dynamic fashion. Therefore, NBI may be an ideal method for examining the angiogenesis of the tumor. In our study, adenocarcinoma made up only 20.6% of all lung cancers. This is different from the results obtained in the studies of Zaric et al. and Miyazu et al, who observed that SCC and adenocarcinoma accounted for the majority of cancers (19, 21). The pathological mechanisms resulting in different vascular patterns in different histological types are still unclear, and future work will be expected to focus on the mechanisms that underlie the different vascular patterns observed *via* NBI.

There were some limitations in this study. Most notably, the patients selected for this study showed obvious symptoms because they were selected when they visited a physician, which explains why cases of invasive cancer accounted for the vast majority of lung cancers in our study, and also explains the fact that pre-invasive lesions (CIS, moderate and severe atypical hyperplasia and squamous metaplasia) were fewer. Further research will be needed to investigate the vascular patterns of NBI of these lesions. In our study, the number of adenocarcinoma was also small, so the vascular patterns of adenocarcinoma need to be further studied.

## Conclusion

This study shows that NBI is a safe and effective technology that is very valuable in the diagnosis of central lung cancer. Vascular patterns as visualized by NBI also demonstrate a certain predictive role for the histological types of lung cancer.

## Data availability statement

The original contributions presented in the study are included in the article/**Supplementary Material**. Further inquiries can be directed to the corresponding author.

## Ethics statement

The studies involving human participants were reviewed and approved by the ethics committee of the First Affiliated Hospital of Bengbu Medical College, Approval Number 2015-017. The patients/participants provided their written informed consent to participate in this study.

## Author contributions

WL designed the study. WL, JZ, RL, BG and YS performed the study. YO conducted the pathological analysis. ZJ and WL drafted the manuscript. All authors read and approved the final manuscript.

## Funding

This work was funded by Academic Subsidy Project for Top Talents in Disciplines of Colleges of Provincial Education Department (gxbjZD16), Key Research and Development Projects in Anhui Province For A (201904a07020079) and the Anhui science and technology development fund projects guided by China government in 2021(2020b07030008).

## Acknowledgments

We thank Dr. Kerry Mills for copy editing of the manuscript.

## Conflict of interest

The authors declare that the research was conducted in the absence of any commercial or financial relationships that could be construed as a potential conflict of interest.

## Publisher’s note

All claims expressed in this article are solely those of the authors and do not necessarily represent those of their affiliated organizations, or those of the publisher, the editors and the reviewers. Any product that may be evaluated in this article, or claim that may be made by its manufacturer, is not guaranteed or endorsed by the publisher.
