# Supplementary material for: The value of narrow-band imaging bronchoscopy in diagnosing central lung cancer
Source: Front Oncol. 2022 Sep 16;12:998770. doi: 10.3389/fonc.2022.998770 (PMC9524255; doi:10.3389/fonc.2022.998770)
Supplement: Supplementary file 1 [file DataSheet_1.docx]

Supplementary Material

**Supplementary Table 1：Assignment of variables**

| **Variable symbol** | **Variable content** | **Assignment method** |
| --- | --- | --- |
| X1 | Gender | Male=1, female=2 |
| X2 | Age (year) | 0～40=0，41～60=1，61～80=2，81～=3 |
| X3 | Smoking status | No smoking=0, smoking=1 |
| X4 | Vascular patterns under NBI | Abrupt-ending=1,Tortuous=2, Dotted=3 |
| Y | Histological types of lung cancer | Adenocarcinoma=1, SCC=2, SCLC =3 |

**Supplementary Table 2：Results of univariate polynomial Logistic analysis is (parameter estimation)**

| Histological types  of lung cancer ^A^ | Variable symbol | B | P | Exp(B) |
| --- | --- | --- | --- | --- |
| SCC | X1 | -2.779 | <0.001 | 0.062 |
|  | X2 | 0.576 | 0.006 | 1.779 |
|  | X3 | 2.995 | <0.001 | 19.204 |
|  | X41 | 3.467 | <0.001 | 32.051 |
|  | X42 | 2.749 | <0.001 | 15.620 |
|  | X43 | 0^B^ |  |  |

Note: A: The reference category is adenocarcinoma; B: It is set to 0, because of this parameter is redundant.

**Supplementary Table 3：Results of univariate polynomial Logistic analysis is (parameter estimation)**

| Histological types  of lung cancer ^A^ | Variable symbol | B | P | Exp(B) |
| --- | --- | --- | --- | --- |
| SCLC | X1 | -0.838 | 0.002 | 0.432 |
|  | X2 | 0.396 | 0.068 | 1.486 |
|  | X3 | 2.092 | <0.001 | 8.104 |
|  | X41 | 3.009 | <0.001 | 20.263 |
|  | X42 | 4.707 | <0.001 | 110.688 |
|  | X43 | 0^B^ |  |  |

Note: A: The reference category is adenocarcinoma; B: It is set to 0, because of this parameter is redundant.

**Supplementary Table 4：Results of multivariate polynomial Logistic analysis is (parameter estimation)**

| Histological types  of lung cancer ^A^ | Variable symbol | B | P | Exp(B) |
| --- | --- | --- | --- | --- |
| SCC | intercept | -0.971 | 0.377 |  |
|  | X1 | -0.993 | 0.078 | 0.371 |
|  | X2 | -0.435 | 0.169 | 0.647 |
|  | X3 | 2.820 | <0.001 | 16.771 |
|  | X41 | 3.589 | <0.001 | 36.196 |
|  | X42 | 2.925 | <0.001 | 18.638 |
|  | X43 | 0^B^ |  |  |

Note: A: The reference category is adenocarcinoma; B: It is set to 0, because of this parameter is redundant.

**Supplementary Table 5：Results of multivariate polynomial Logistic analysis is (parameter estimation)**

| Histological types  of lung cancer ^A^ | Variable symbol | B | P | Exp(B) |
| --- | --- | --- | --- | --- |
| SCLC | intercept | -3.479 | 0.003 |  |
|  | X1 | 0.956 | 0.064 | 2.601 |
|  | X2 | -0.742 | 0.026 | 0.476 |
|  | X3 | 2.913 | <0.001 | 18.411 |
|  | X41 | 3.158 | <0.001 | 23.526 |
|  | X42 | 4.916 | <0.001 | 136.415 |
|  | X43 | 0^B^ |  |  |

Note: A: The reference category is adenocarcinoma; B: It is set to 0, because of this parameter is redundant.
